# Supplementary material for: Non-Classical HLA Determinants of the Clinical Response after Autologous Stem Cell Transplantation for Systemic Sclerosis
Source: Int J Mol Sci. 2022 Jun 29;23(13):7223. doi: 10.3390/ijms23137223 (PMC9266677; doi:10.3390/ijms23137223)
Supplement: Supplementary file 1 [file ijms-23-07223-s001.zip › ijms-1740928-supplementary final version.pdf]

Table S1: Pair-wise analysis of the HLA class I (-A.-B and -C) genotype frequencies genotype frequencies within the Systemic Sclerosis patients, according to the observed clinical response (responder or non-responder) at 12-months after autologous hematopoietic stem cell transplantation.

**HLA alleles**	**Frequency**		**P-value**	**HLA alleles**	**Frequency**		**P-value**
HLA-A	**R (n=32)**	**NR (n=13)**	**R vs NR**	***HLA-B***	**R (n=32)**	**NR (n=13)**	**R vs NR**
*A*01:01*	0.16	0.15	1.000	*B*38:02*	0.02	0.04	0.497
*A*02:01*	0.22	0.23	0.901	*B*39:01*	0.03	0.00	1.000
*A*02:03*	0.00	0.04	0.289	*B*39:10*	0.00	0.04	0.289
*A*02:05*	0.00	0.08	0.081	*B*40:01*	0.02	0.00	1.000
*A*02:07*	0.02	0.04	0.497	*B*40:02*	0.03	0.00	1.000
*A*03:01*	0.06	0.04	1.000	*B*42:01*	0.00	0.04	0.289
*A*11:01*	0.08	0.04	0.668	*B*42:02*	0.02	0.00	1.000
*A*23:01*	0.06	0.00	0.320	*B*44:02*	0.02	0.04	0.497
*A*23:17*	0.02	0.04	0.497	*B*44:03*	0.02	0.00	1.000
*A*24:02*	0.11	0.12	1.000	*B*44:05*	0.02	0.00	1.000
*A*26:01*	0.03	0.04	1.000	*B*45:01*	0.06	0.04	1.000
*A*29:02*	0.02	0.00	1.000	*B*46:01*	0.02	0.00	1.000
*A*30:01*	0.05	0.12	0.350	*B*49:01*	0.06	0.04	1.000
*A*32:01*	0.03	0.00	1.000	*B*50:01*	0.02	0.00	1.000
*A*33:01*	0.02	0.00	1.000	*B*51:01*	0.05	0.08	0.624
*A*33:03*	0.03	0.00	1.000	*B*51:07*	0.02	0.00	1.000
*A*34:02*	0.03	0.00	1.000	*B*55:01*	0.05	0.00	0.554
*A*66:01*	0.02	0.00	1.000	*B*56:01*	0.00	0.04	0.289
*A*68:01*	0.03	0.04	1.000	*B*57:03*	0.00	0.04	0.289
*A*68:02*	0.02	0.04	0.497	*B*58:01*	0.02	0.04	0.497
*A*74:01*	0.02	0.00	1.000	***HLA-C***	**R (n=32)**	**NR (n=13)**	**R vs NR**
HLA-B	**R (n=32)**	**NR (n=13)**	**R vs NR**	*C*01:02*	0.02	0.04	0.497
*B*07:02*	0.08	0.08	1.000	*C*02:02*	0.09	0.04	0.668
*B*08:01*	0.05	0.04	1.000	*C*02:10*	0.02	0.04	0.497
*B*13:01*	0.00	0.04	0.289	*C*03:02*	0.02	0.00	1.000
*B*13:02*	0.03	0.00	1.000	*C*03:03*	0.09	0.00	0.183
*B*14:02*	0.02	0.08	0.199	*C*03:04*	0.05	0.08	0.624
*B*15:01*	0.09	0.04	0.668	*C*04:01*	0.11	0.15	0.723
*B*15:03*	0.00	0.04	0.289	*C*04:03*	0.02	0.00	1.000
*B*15:10*	0.02	0.00	1.000	*C*05:01*	0.03	0.04	1.000
*B*15:17*	0.02	0.00	1.000	*C*06:02*	0.08	0.08	1.000
*B*15:25*	0.02	0.00	1.000	*C*07:01*	0.13	0.12	1.000
*B*18:01*	0.08	0.04	0.668	*C*07:02*	0.11	0.12	1.000
*B*27:05*	0.05	0.00	0.554	*C*07:18*	0.02	0.00	1.000
*B*35:01*	0.00	0.12	**0.022***	*C*08:02*	0.02	0.08	0.199
*B*35:02*	0.06	0.04	1.000	*C*12:03*	0.09	0.04	0.668
*B*35:03*	0.02	0.04	0.497	*C*14:02*	0.06	0.08	1.000
*B*37:01*	0.03	0.04	1.000	*C*16:01*	0.03	0.04	1.000
*B*38:01*	0.02	0.00	1.000	*C*16:02*	0.00	0.04	0.289
*** not significant after Benjamini Hocherberg Correction (FDR)**				*C*17:01*	0.03	0.04	1.000

R :responder patients, NR : non-responder patients

Due to missing DNA sample, one non-responder patient was not typed for all HLA loci. Therefore, a total of 13 out of 14 non-responder patients were analyzed at 12 months.

Table S2: Pair-wise analysis of the HLA class I (-A.-B and -C) genotype frequencies genotype frequencies within the -Systemic Sclerosis patients, according to the observed clinical response (responder or non-responder) at 24-months after autologous hematopoietic stem cell transplantation.

HLA alleles	Frequency		P-value
HLA-A	**R (n=33)**	**NR (n=12)**	**R vs NR**
*A*01:01*	0.17	0.13	0.752
*A*02:01*	0.20	0.29	0.339
*A*02:03*	0.00	0.04	0.267
*A*02:05*	0.00	0.08	0.069
*A*02:07*	0.03	0.00	1.000
*A*03:01*	0.08	0.00	0.319
*A*11:01*	0.08	0.04	1.000
*A*23:01*	0.06	0.00	0.570
*A*23:17*	0.02	0.04	0.464
*A*24:02*	0.11	0.13	0.723
*A*26:01*	0.03	0.04	1.000
*A*29:02*	0.02	0.00	1.000
*A*30:01*	0.05	0.13	0.336
*A*32:01*	0.03	0.00	1.000
*A*33:01*	0.02	0.00	1.000
*A*33:03*	0.03	0.00	1.000
*A*34:02*	0.03	0.00	1.000
*A*66:01*	0.02	0.00	1.000
*A*68:01*	0.03	0.04	1.000
*A*68:02*	0.02	0.04	0.464
*A*74:01*	0.02	0.00	1.000
HLA-B	**R (n=33)**	**NR (n=12)**	**R vs NR**
*B*07:02*	0.09	0.04	0.670
*B*08:01*	0.05	0.04	1.000
*B*13:01*	0.00	0.04	0.267
*B*13:02*	0.03	0.00	1.000
*B*14:02*	0.03	0.04	1.000
*B*15:01*	0.09	0.04	0.670
*B*15:03*	0.00	0.04	0.267
*B*15:10*	0.02	0.00	1.000
*B*15:17*	0.02	0.00	1.000
*B*15:25*	0.02	0.00	1.000
*B*18:01*	0.08	0.04	1.000
*B*27:05*	0.05	0.00	0.562
*B*35:01*	0.00	0.13	**0.017***
*B*35:02*	0.06	0.04	1.000
*B*35:03*	0.02	0.04	0.464
*B*37:01*	0.05	0.00	0.562
*B*38:01*	0.02	0.00	1.000
*B*38:02*	0.02	0.04	0.464
*B*39:01*	0.03	0.00	1.000
*B*39:10*	0.00	0.04	0.267
*B*40:01*	0.02	0.04	0.267
*B*40:02*	0.03	0.00	1.000
*B*42:01*	0.00	0.04	0.267
*B*42:02*	0.02	0.00	1.000
*B*44:02*	0.02	0.08	0.069
*B*44:03*	0.02	0.00	1.000
*B*44:05*	0.02	0.00	1.000
*B*45:01*	0.06	0.04	1.000
*B*46:01*	0.02	0.00	1.000
*B*49:01*	0.06	0.04	1.000
*B*50:01*	0.02	0.00	1.000
*B*51:01*	0.05	0.04	1.000
*B*51:07*	0.02	0.00	1.000
*B*55:01*	0.05	0.00	0.562
*B*56:01*	0.00	0.04	0.267
*B*57:03*	0.00	0.04	0.267
*B*58:01*	0.02	0.04	0.464
HLA-C	**R (n=33)**	**NR (n=12)**	**R vs NR**
*C*01:02*	0.02	0.04	0.464
*C*02:02*	0.09	0.04	0.670
*C*02:10*	0.02	0.04	0.464
*C*03:02*	0.02	0.00	1.000
*C*03:03*	0.09	0.00	0.187
*C*03:04*	0.03	0.13	0.116
*C*04:01*	0.11	0.17	0.475
*C*04:03*	0.02	0.00	1.000
*C*05:01*	0.02	0.08	0.172
*C*06:02*	0.09	0.04	0.670
*C*07:01*	0.12	0.13	1.000
*C*07:02*	0.12	0.08	1.000
*C*07:18*	0.02	0.00	1.000
*C*08:02*	0.03	0.04	1.000
*C*12:03*	0.09	0.04	0.670
*C*14:02*	0.08	0.04	1.000
*C*16:01*	0.03	0.04	1.000
*C*16:02*	0.00	0.04	0.267
*C*17:01*	0.03	0.04	1.000

*** not significant after Benjamini Hocherberg Correction (FDR)**

R: responder patients, NR: non-responder patients

Due to missing DNA sample, one non-responder patient was not typed for all HLA loci. Therefore, a total of 12 out of 13 non-responder patients were analyzed at 24 months.

Table S3: Linear logistic regression to evaluate relationship between independent variables and mRSS at 3 months after AHSCT.

	**mRSS at 3 months**		
Predictors	*Estimates*	*CI*	*p*
(Intercept)	9.36	-6.78 – 25.50	0.247
Sex [M]	-3.33	-9.08 – 2.43	0.248
Age at transplant	0.26	0.02 – 0.50	**0.035**
Disease duration	-1.88	-4.05 – 0.29	0.087
ILD on HRCT BL [Yes]	-6.95	-16.17 – 2.26	0.134
Antic Scl70 BL [Positive]	1.00	-5.08 – 7.09	0.740
HLA-C rs2395471 [AA vs Others]	7.41	0.33 – 14.49	**0.041**
Origin [European&North African vs Others]	7.53	0.88 – 14.19	**0.028**
Observations	41		
R^2 / R^2adjusted	0.411 / 0.286		

BL: Baseline; CI : confidence interval; HRCT: High-resolution computed tomography; ILD : Interstitial Lung Disease ; p : p-value;

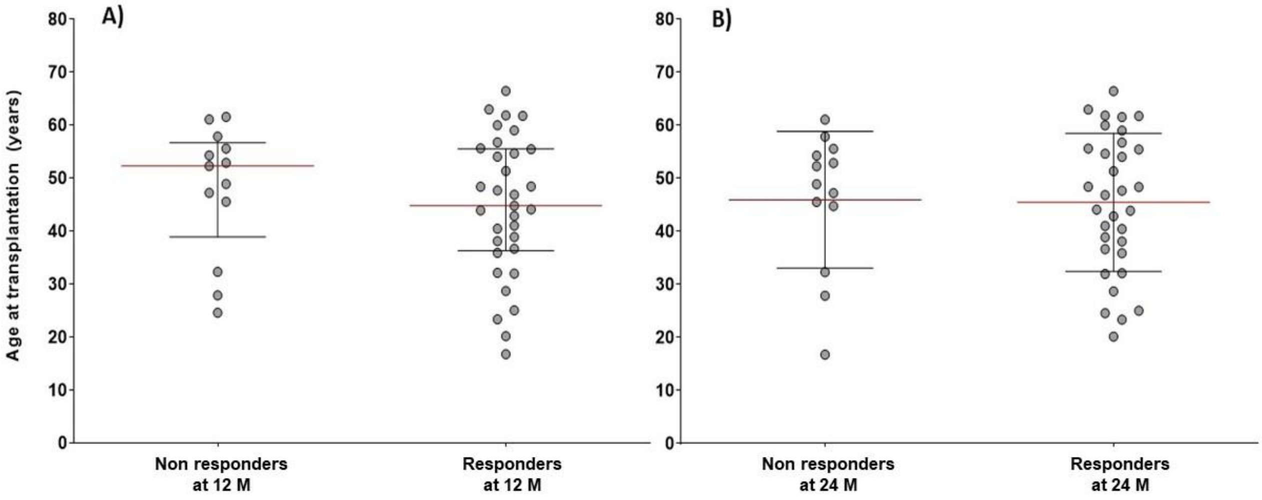

Figure S1. Age at transplantation of the 46-Systemic Sclerosis patients according to the observed clinical response (responder or non-responder) at 12-months and 24-months after autologous hematopoietic stem cell transplantation. Each patient is represented by a grey circle. Patients are separated into two groups, responders and non-responders to ASCT **A)** at 12 months, **B)** at 24 months. The two groups are compared for age at transplantation by Mann Whitney test. Age medians are represented by a red line. Interquartile ranges are represented by a black line.
